# Targeted genomic analysis of cutaneous T cell lymphomas identifies a subset with aggressive clinicopathological features

**DOI:** 10.1038/s41408-020-00380-5

**Published:** 2020-11-09

**Authors:** Kimon V. Argyropoulos, Melissa Pulitzer, Francesco Maura, Abhinita Mohanty, Patrizia Mondello, Steven M. Horwitz, Patricia Myskowski, Alison Moskowitz, Ahmet Dogan, Christiane Querfeld, Franck Rapaport, Marina Siakantaris, Peter C. Louis, Natasha Galasso, Marcel R. M. van den Brink, M. Lia Palomba

**Affiliations:** 1 Department of Immunology, Memorial Sloan Kettering Cancer Center York, New York, NY USA; 2 National and Kapodistrian University of Athens, Athens, Greece; 3 Department of Pathology, Memorial Sloan Kettering Cancer Center York, New York, NY USA; 4 Department of Medicine, Memorial Sloan Kettering Cancer Center York, New York, NY USA; 5 City of Hope Hospital, Duarte, CA USA; 6 Center for Hematologic Malignancies, Memorial Sloan Kettering Cancer Center York, New York, NY USA; 7 Department of Biomedical Informatics, Vanderbilt University Medical Center, Nashville, TN USA

**Keywords:** Translational research, Cancer genomics

## Dear Editor,

Cutaneous T-cell Lymphomas (CTCLs) have been shown to have a complex mutational landscape. Despite the availability of molecular data it is unclear whether they have any diagnostic and prognostic utility. In this study, we performed targeted sequencing for 585 genes that are frequently mutated in solid and hematological malignancies (MSKCC HemePACT) in 77 CTCL samples, including lesions from early Mycosis Fungoides (eMF) (*n* = 21), advanced Mycosis Fungoides/large cell transformation (aMF–LCT) (*n* = 15), Sézary syndrome (SS) (*n* = 17), CD30+ Lymphoproliferative Disorders (CD30+LPD) (*n* = 12), γδCTCLs (*n* = 5) and other rare CTCLs (*n* = 7). We identified genetic alterations in 358 genes with eMF lesions showing the lowest mutational burden, while aMF lesions showed the highest mutational burden among all subtypes. C>T transitions were the predominant substitution among all subsets with the exception of eMF lesions. Although there was remarkable pathway heterogeneity, all CTCL histological subsets carried mutations in the GPCR/RTK/MAPK signaling pathway. Only four genes were recurrently mutated in more than 10% of CTCLs: CDKN2A/B, PCLO, FAT1, and TP53. We identified that the presence of mutations in at least one of those genes defined a CTCL subset with increased tumor burden, aggressive immunopathological features, and dismal prognosis.

CTCLs constitute a heterogeneous group of lymphoproliferative neoplasms, which differ widely in terms of biology, histopathology, and clinical presentation^1,2^. Whole-genome, whole-exome, and targeted sequencing approaches have identified a complex mutational landscape, affecting genes involved in immune-synapse signaling, cell-cycle regulation, and epigenetic modulation^3–11^. Most studies thus far have focused on MF and SS, while the molecular features of rarer CTCLs are not fully elucidated and there is a lack of meaningful molecular signatures that could be used in the clinical setting for all CTCLs. The goal of this study was (1) to characterize concomitantly multiple CTCL subsets and identify the predominant genetic events that characterize each entity and (2) to identify clinically meaningful molecular CTCL subgroups.

**Fig. 1:**
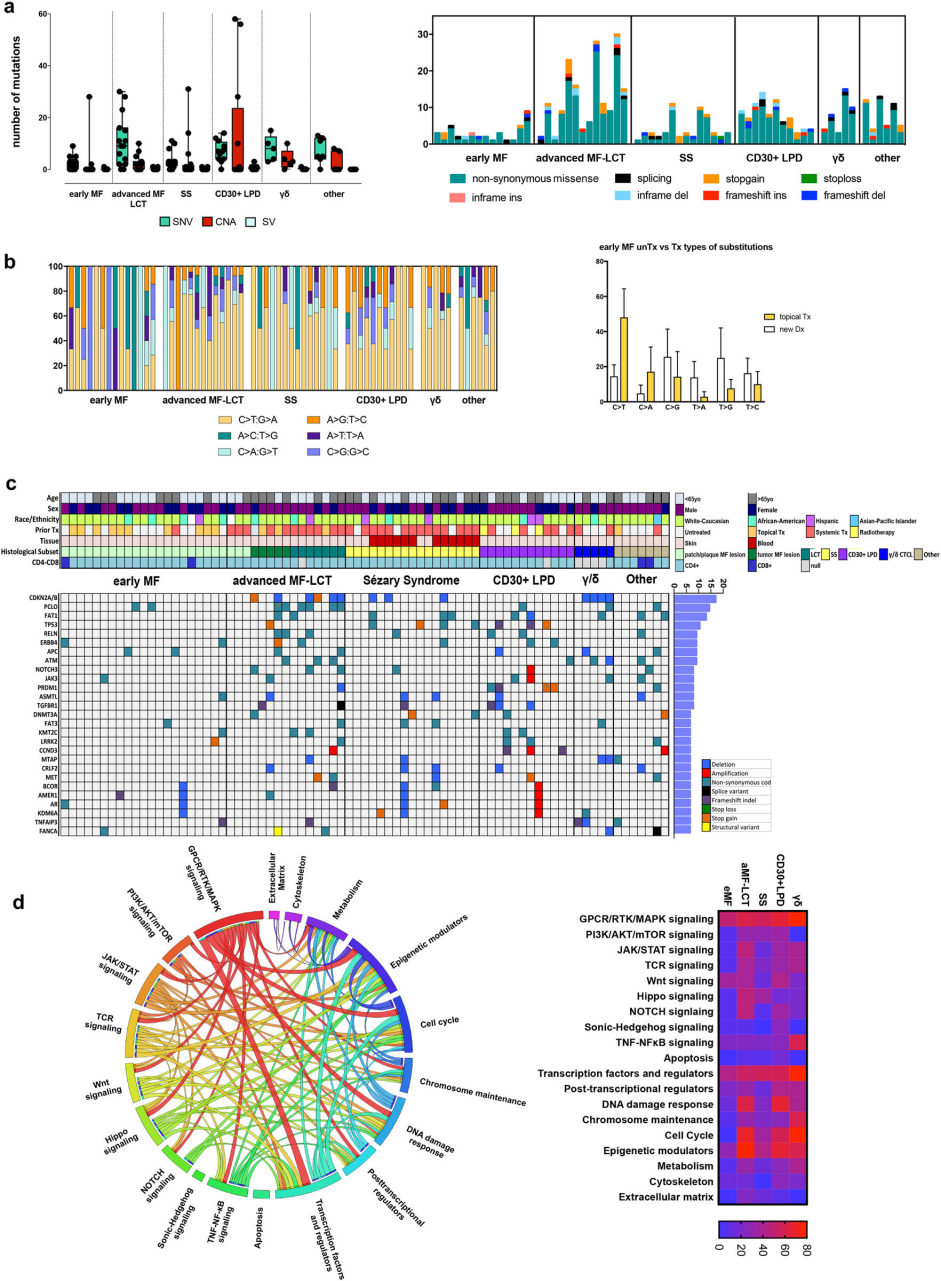
Genomic alterations and biologic pathways in CTCL. **a** Illustration of SNV, CNA, and SV load (left) and representation of the identified SNV types (right) in different CTCL subsets. **b** Base substitutions identified in different CTCL subsets (left) and base substitutions in newly diagnosed/untreated versus topically treated eMF lesions (right). **c** Oncoprint of recurrent (>5%) alterations incorporating SNVs, CNAs, and SVs. The first seven rows represent corresponding demographic and clinicopathologic data. **d** CIRCOS plot illustrating 19 recurrently mutated pathways and their cooccurence in CTCLs (left), as well as the frequency of mutated pathways in different CTCL subsets (right).

We performed targeted sequencing for 585 genes frequently mutated in solid and hematological malignancies (MSKCC HemePACT^12^) in 77 CTCL samples with paired germline control samples when available (*n* = 43). All patients had previously signed informed consent to have their specimen used for research purposes, in accordance with the Declaration of Helsinki and approval by the Memorial Sloan Kettering Cancer Center Institutional Review Board. Figure 1c and Supplementary Table 1 summarize patient demographics and clinicopathological characteristics. Germline and neoplastic material was sequenced on a HiSeq2500 Illumina instrument. Detailed description of mutation analysis and histological/immunophenotypic analysis can be found in the Supplementary material.

We identified single nucleotide variants (SNVs), copy number alterations (CNAs) and structural variants (SVs) in 358 genes, with 537X sequencing depth being the median for all filtered somatic variants (Supplementary Table 2). Early MF (eMF) lesions showed a low mutational burden, with 33.3% of cases having no detectable alterations (Fig. 1a). Advanced MF and large cell transformed (aMF-LCT) lesions showed the highest mutational burden among all subtypes. Interestingly, 4 out of 12 CD30+ lymphoproliferative disease (CD30+ LPD) samples exhibited multi-chromosomal CNAs. No difference in mutational burden or type of substitution was observed when data were analyzed according to sex, age, or race/ethnicity (Supplementary Fig. 1a, b, c). C>T transitions were the most prominent events among all CTCLs (Fig. 1b). In eMF lesions, we observed a bias towards C>T transitions primarily in samples that had received prior topical treatment rather than newly diagnosed/treatment naïve samples. This was not related to an inherent aggressiveness of the treated lesions, as the two eMF groups had a similar distribution of stage IA and IB cases (Supplementary Fig. 1d) and no difference in variant allele frequency (VAF) numbers, which reflect the tumor content of the lesion (Supplementary Fig. 1e, f). Further mutational signature analysis showed that the single base substitution (SBS) signature related to ultraviolet (UV) damage represented 34.8% of events (Supplementary Fig. 2a, b). Although 44.1% of our specimen were from patients that had received systemic therapy, no chemotherapy-related SBS signatures were identified.

Forty-six genes showed recurrent alterations in more than 5% of CTCLs (Fig. 1c). Regardless of CTCL histology, alterations in more than 10% of the cohort were identified in CDKN2A and B, PCLO, FAT1, and TP53 (Supplementary Fig. 3). CDKN2A and B alterations occurred in 16.9% of CTCL (*n* = 13, SNVs, *n* = 2; gene deletions, *n* = 11). PCLO exhibited the highest frequency of SNVs (14.9%, *n* = 11 sample, *n* = 15 mutations), which were all missense mutations, with one sample carrying an additional stop codon gain mutation. FAT1 harbored somatic alterations in 13% of CTCL (*n* = 10, SNVs, *n* = 9; gene deletions, *n* = 1), while its homolog FAT3 showed missense SNV in 6.5% of CTCL (*n* = 5). TP53 was mutated in 10.4% (*n* = 8) of all samples, harboring missense variants (*n* = 3), stop gain variants (*n* = 3), and frameshift deletions (*n* = 2). Other recurrently mutated genes (frequency > 7%) included RELN, ERBB4, APC, ATM, NOTCH3, JAK3, PRDM1, ASMTL, and TGFBR1. Despite the heterogeneity of the genomic landscape in CTCL, and although no recurrently mutated gene was specific for a CTCL subtype, we observed differential mutated gene distribution between CTCL subtypes. CDKN2A or B alterations were present in aMF-LCT, SS, and γδCTCL samples (40%, 17.6%, and 80%, respectively), and absent from eMF, CD30+LPD, and other rare CTCL subsets. Moreover, although PCLO was mutated in 40% of aMF-LCT, it was not mutated in SS, while the majority of TP53 mutations occurred in SS and CD30+LPD (23.5% and 25%, respectively). CD30+LPD showed recurrent (25%) alterations in TGFBR1, PRDM1, CCND3, PTCH1, and POLE. Finally, γδCTCL showed recurrent (40%) alterations in ATM, MTAP, TNFAIP3, SOCS1, and SMC3. Pathway analysis identified at least 19 distinct pathways involved in CTCLs with (a) GPCR/RTK/MAPK signaling molecules, (b) transcription factors and regulators, and (c) epigenetic modulators being mutated in more than 50% of the samples. GPCR/RTK/MAPK signaling was within the top three mutated pathways involved in all CTCL histological subtypes. Cell cycle-related alterations never occurred in eMF patches or plaques, yet they were highly prevalent in tumor MF or LCT lesions, suggesting that the acquisition of such events might contribute to tumor progression or LCF of eMF lesions (Fig. 1d).

**Fig. 2:**
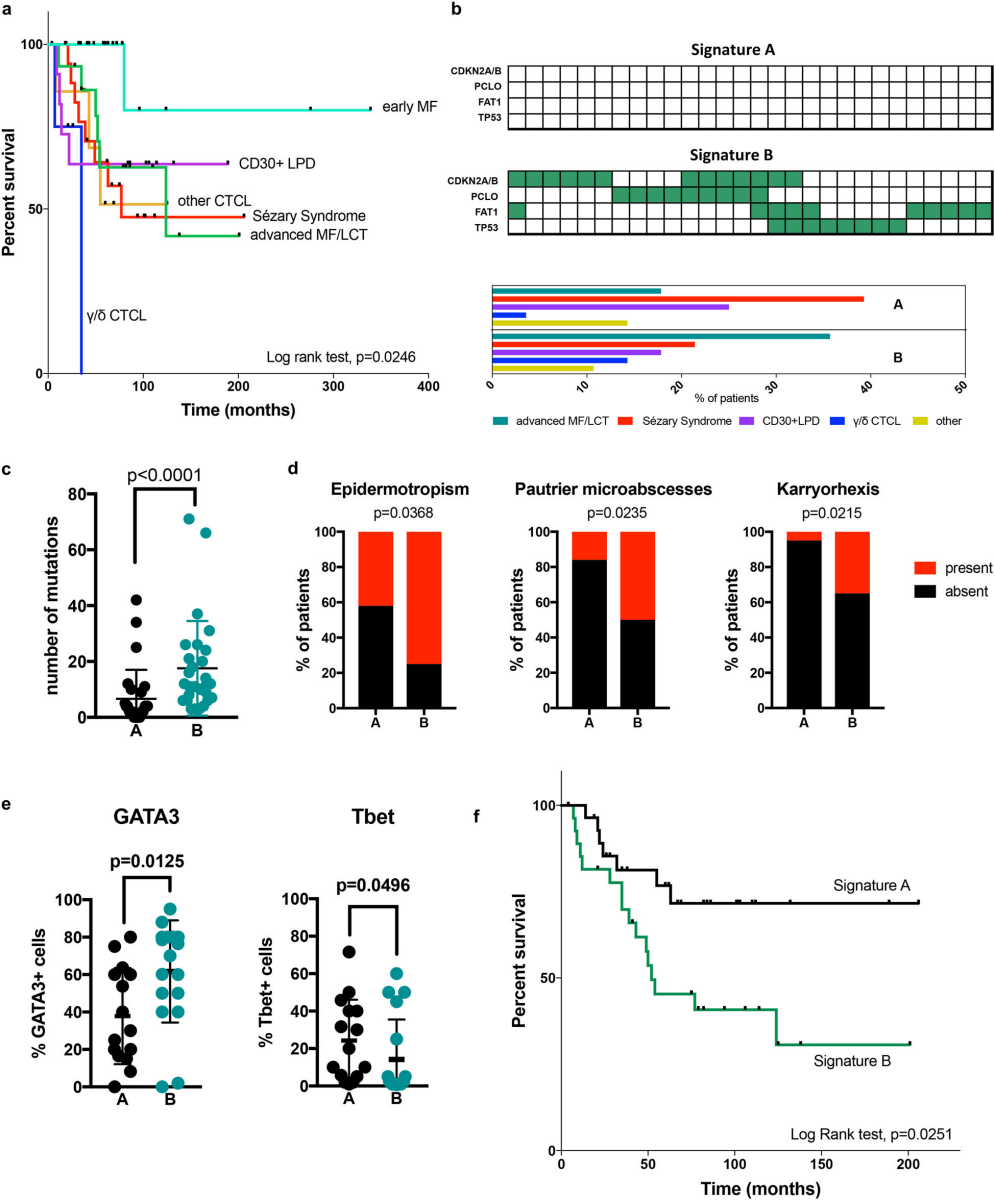
Association between genomic signatures and outcome among subtypes of CTCL. **a** Retrospective analysis of overall survival in CTCL subsets classified according to the WHO classification system (log rank test). **b** Definition of signature A (no alterations) and B (at least one genetic alteration) according to the status of the CDKN2A/B, PCLO, FAT1, and TP53 genes with the differential distribution of CTCL subsets between molecular signatures (eMF samples are excluded). **c** Mutational load (Mann–Whitney test). **d** Occurrence of epidermotropism, Pautrier microabscess formation and karryorhexis in signatures A and B (Chi-square test). **e** Quantification of the frequency of GATA3 and Tbet-positive malignant T cells between molecular signatures (Mann–Whitney test). **f** Overall univariate survival analysis in non-eMF patients based on molecular signature status (log rank test).

Since eMF was mutationally silent and carried a significantly more indolent course compared to all other CTCL subsets in a retrospective analysis of overall survival (Fig. 2a), we focused on identifying molecular events that can discriminate non-eMF CTCLs with aggressive histopatholgical and clinical features, which are indistinguishable under the current WHO classification system. Due to the high number of non-recurrent alterations, unsupervised clustering analysis failed to classify samples into large enough groups that could be further analyzed for clinicopathological correlations (Supplementary Fig. 4). We then classified non-eMF CTCLs based on the absence (Signature A, *n* = 28) or presence (Signature B, *n* = 28) of mutations in at least one of the four most frequently altered genes: CDKN2A/B, PCLO, FAT1, and TP53. CTCL subsets did not show a differential distribution between the two groups (Fig. 2b). Signature B samples had a significantly higher mutational burden, suggesting that this signature could be a surrogate for hypermutated CTCLs (Fig. 2c). From a morphological standpoint signature B samples had a significantly higher presence of epiderrmotropism and Pautrier microabscess formation, while they exhibited a significantly higher incidence of karryorhexis, which is an indicator of higher cell-turnover rate (Fig. 2d). In regard to T cell polarization, despite the heterogeneity in expression and varying coexpression levels of Tbet, GATA3, FoxP3, and Bcl6, malignant T cells showed a Th2-predominant phenotype, as it has been previously described (Supplementary Fig. 5a, b)^13,14^. When broken down by molecular subtype, signature B samples exhibited significantly higher GATA3 and lower Tbet expression (Fig. 2e). No difference was observed in FoxP3 and Bcl6 expressions (data not shown). Finally, overall survival univariate analysis in non-eMF CTCLs showed a significantly more aggressive disease course in patients carrying signature B (Fig. 2f). Multivariate analysis including histological types, age, and prior treatment was also preformed and showed significantly worse outcomes in patients with signature B (Supplementary Fig. 6). This is in par with our immunopathological data, considering the established negative prognostic significance of GATA3 positivity in mature T cell lymphomas^15^. It is worth noting that survival analysis based on the mutation status of for each single gene showed no statistically significant difference compared to WT individuals, with the exception of CDKN2A/B, which only showed a trend for worse outcomes in mutated individuals (Supplementary Fig. 7). Therefore, the concomitant assessment of all four frequently mutated genes appears to be a powerful tool for the prognostication of non-eMF CTCLs, which cannot be provided by the current clinicopathological WHO classification system. Whether all four genes represent true disease drivers and there is biological synergy between them remains to be answered. In summary, CTCLs show few recurrent mutations, which highly overlap between different histological subsets. This study shows that the examination of the mutational status of CDKN2A/B, FAT1, PCLO, and TP53 can be used as a surrogate marker for hypermutated CTCLs with aggressive pathological features and poor prognosis.

## Supplementary information

Supplementary Figures

Supplementary Table 1

Supplementary Table 2

## Data Availability

The data can be made available upon request.
