# Supplementary material for: Targeted genomic analysis of cutaneous T cell lymphomas identifies a subset with aggressive clinicopathological features
Source: Blood Cancer J. 2020 Nov 9;10(11):116. doi: 10.1038/s41408-020-00380-5 (PMC7653963; doi:10.1038/s41408-020-00380-5)
Supplement: Supplementary file 1 — Supplementary Figures [file 41408_2020_380_MOESM1_ESM.pdf]

**Somatic Mutation calling and Copy Number alteration analysis**

We aligned the sequencing reads using BWA MEM. We then called single nucleotide variants (SNVs) using CAVEMAN and short insertions and deletions using PINDEL in each sample compared to a standard uncontaminated normal blood sample sequenced on the same panel. We applied heuristic-based filters (strand bias, unmatched panel of normals) to sort out artifacts following the recommandations of the International Cancer Genome Consortium (ICGC) and the Cancer Genome Pipeline (CGP). The variants were annotated with information from a database of known somatic variants (COSMIC) and a database of known germline events (ExAC). For tumors, which remained unmatched, we removed all variants with an allele frequency below 0.44, a cutoff we set based on the distribution of validated somatic events, and maintained variants previously described as oncogenic or likely oncogenic. Copy number alteration (CNA) data were generated by the GISTIC algorithm.

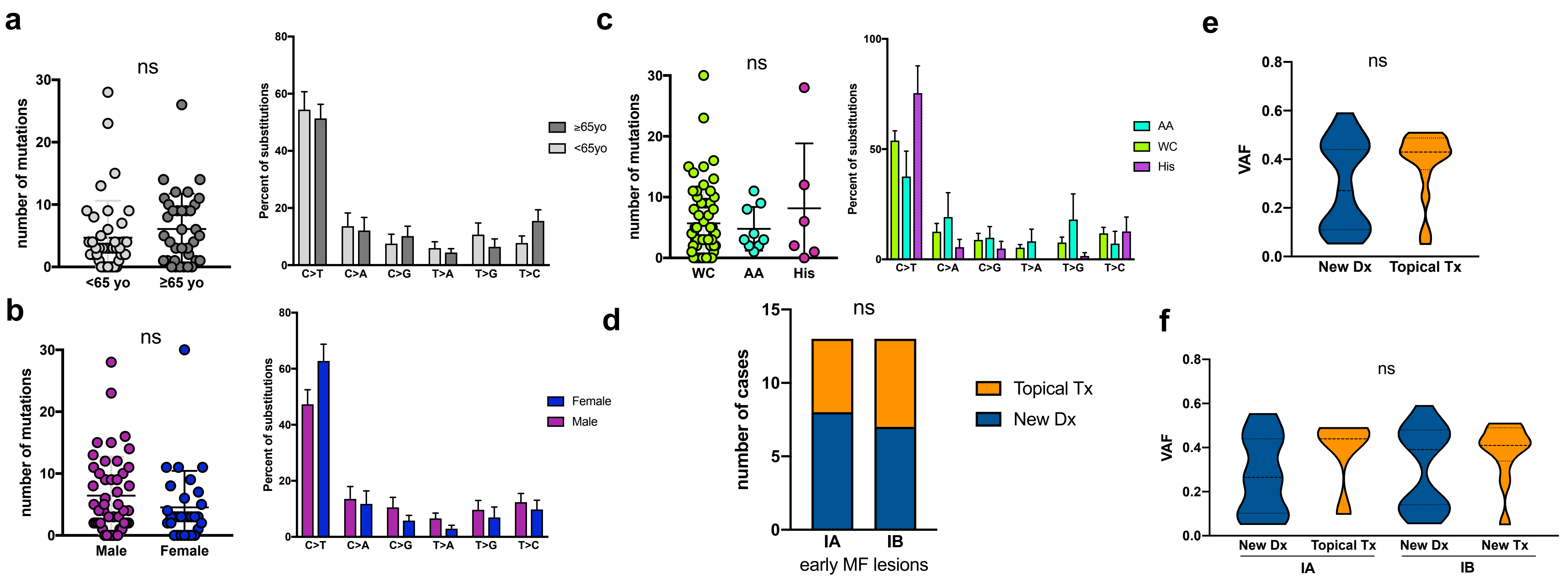


**Supplementary Figure 1**

Number of SNVs and types of substitutions per age (**a,** Mann-Whitney non-parametric test), sex (**b,** Mann-Whitney non-parametric test) and race/ethnicity (**c,** Mann-Whitney non-parametric test) No significant difference was observed when comparing demographic groups. Distribution of stage IA and stage IB cases among eMF patients (**d,** Fisher's exact test), and distribution of VAFs between stage IA vs IB cases (**e,** Mann-Whitney non-parametric test, **f**, Kruskal-Wallis test).

**Mutational signature analysis**

SNV calls from targeted sequencing were subjected to mutational signature fitting, using the previously described R package mmsig (Rustad EH et al. Nature communications. 2020). Only mutational processes previously known to be active in CTCL were included (Maura F et al. Nature communications. 2019; Wang L et al. Nature Genetics. 2015). Cox regression was used to estimate the overall survival hazard ratio for the main clinical and biological features, estimated using multivariate.

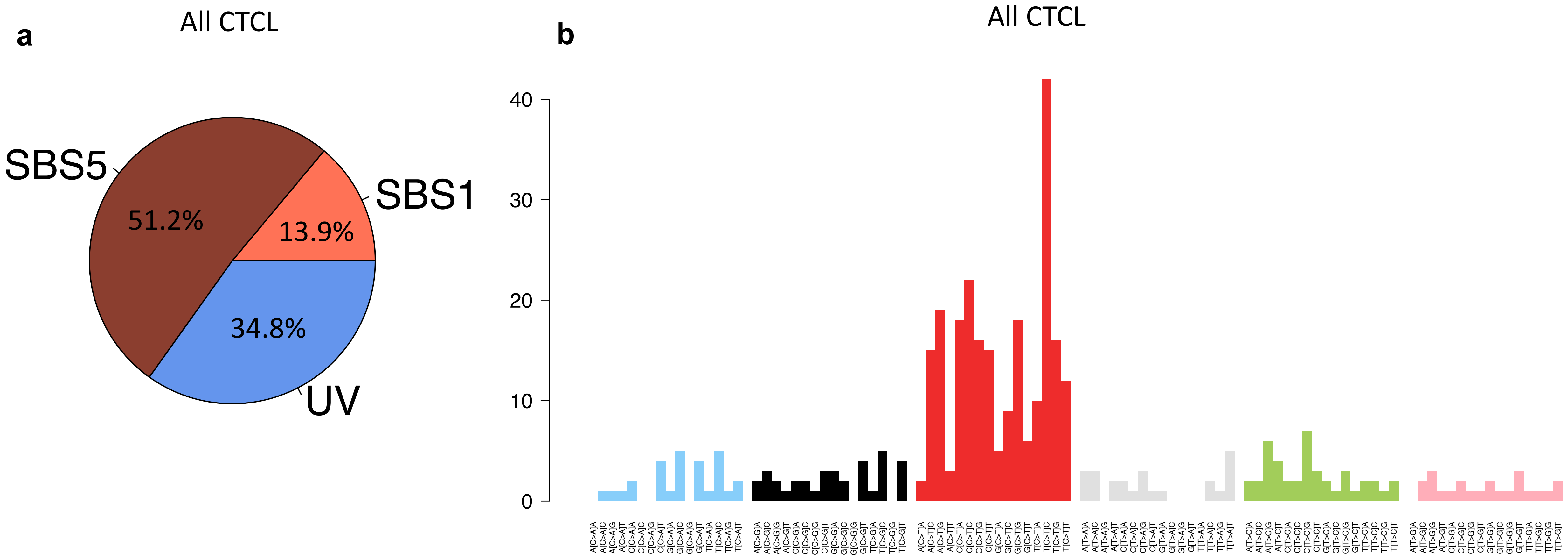


**Supplementary Figure 2**

Mutational signature showing the representation of C>T rich signatures SBS1, SBS5 and UV (SBS7), that have been priorly described in CTCL, among all CTCL specimen (**a**), Mapping of 96-trinucleotide SBS among all CTCL specimen (**b**).

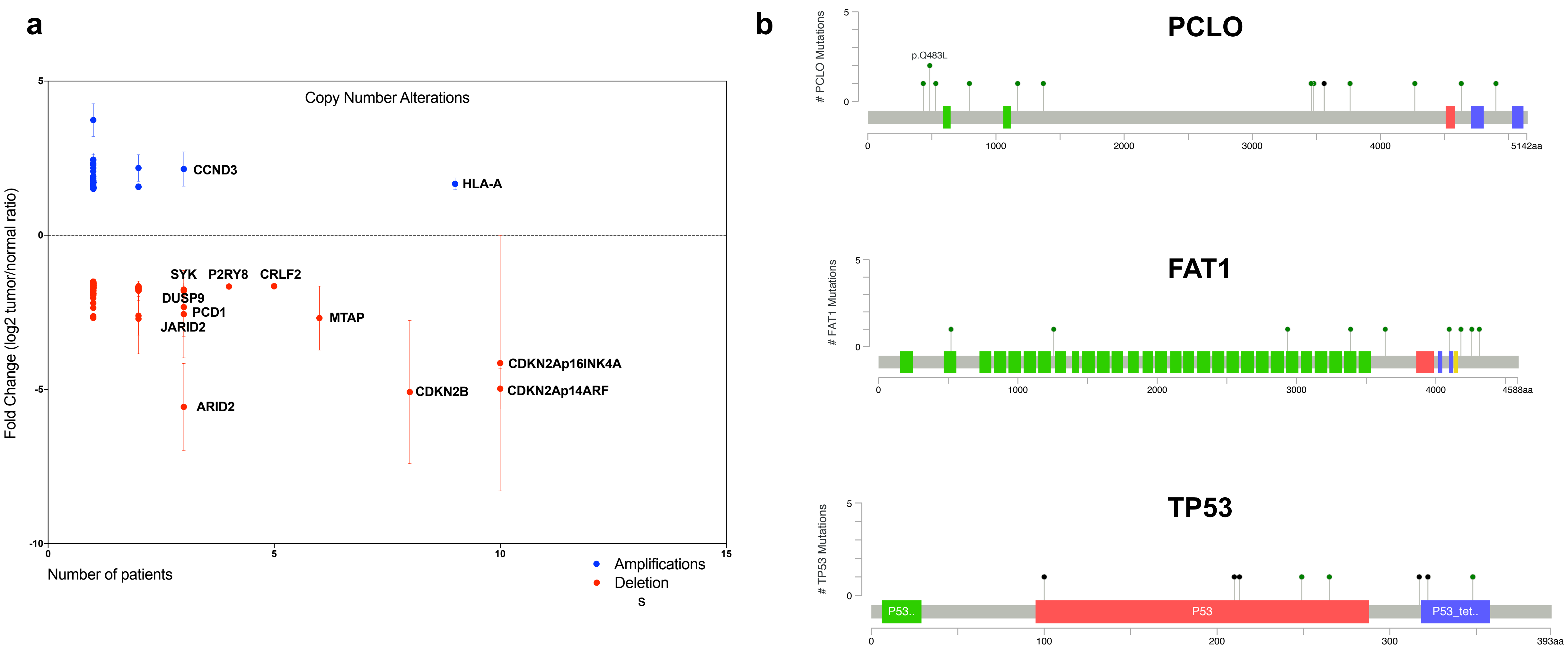


**Supplementary Figure 3**

Plot representing frequent CNAs as a function of the fold change tumor/normal ratio versus the number of patients (**a**). Lollipop plots of the PCLO gene, TP53 gene and FAT1 gene (**b**).

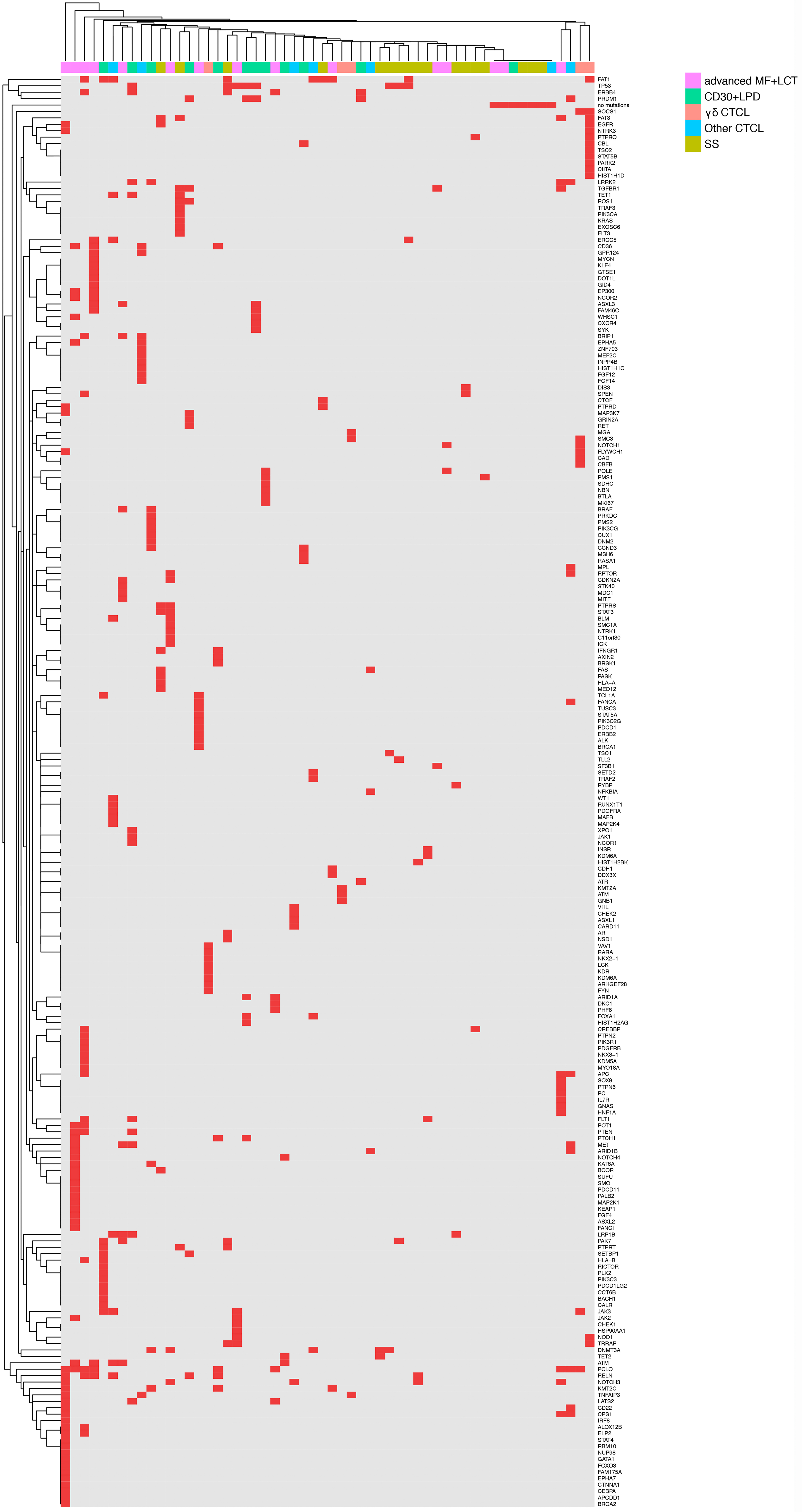


**Supplementary Figure 4**

Oncoprint with unsupervised clustering analysis for all mutations (SNVs and CNAs) identified in all CTCL samples.

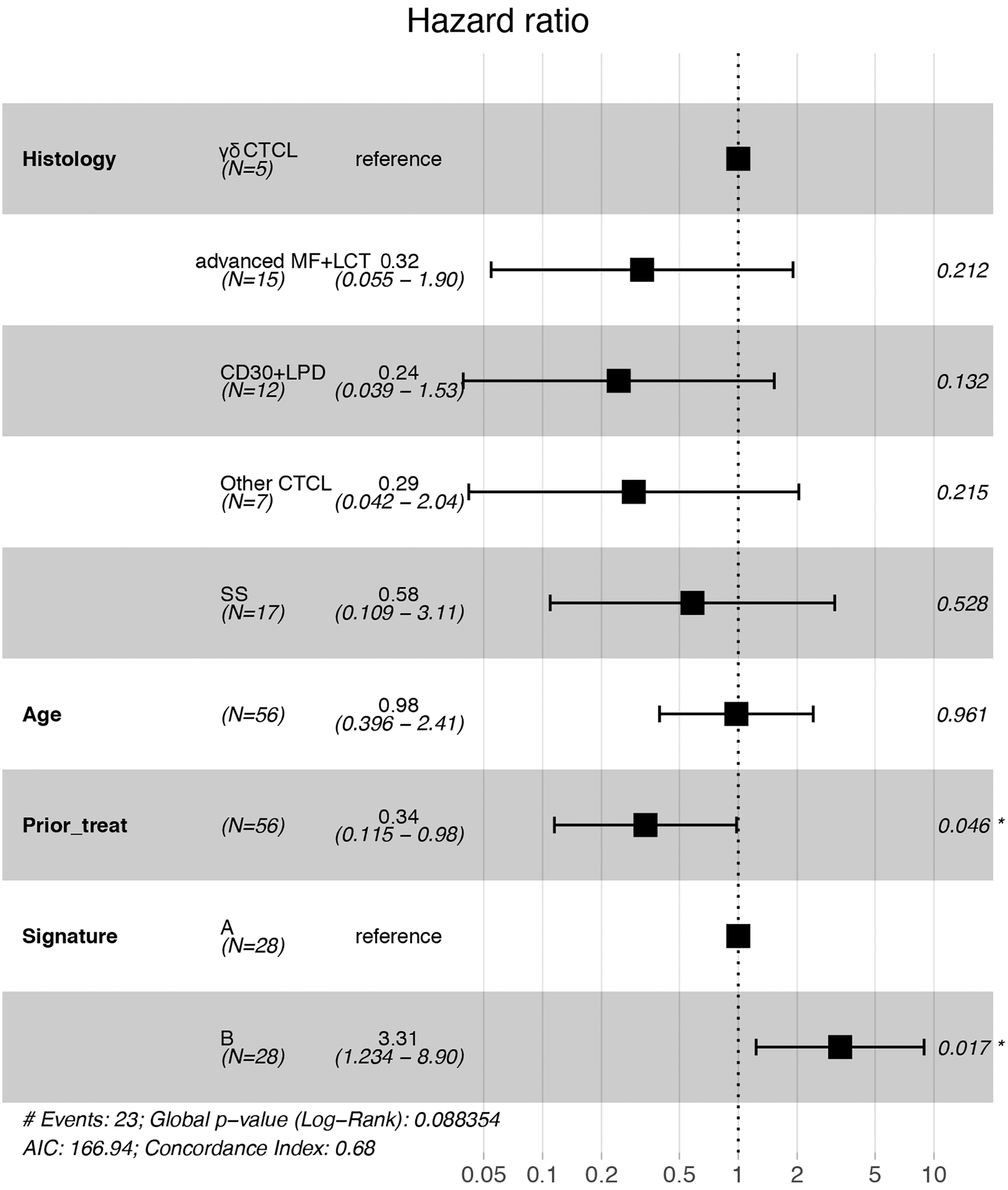


**Supplementary Figure 5**
Cox regression was used to estimate the overall survival hazard ratio for the main clinical, histological and molecular features, estimated using multivariate analysis. Signature B confers a worse prognosis compared to signature A, independent of histology, age and prior treatment. Prior treatment shows a marginally protective effect, though this might stem from the fact that a significant number of “untreated” specimen were retrieved from patients with newly diagnosed aggressive lymphomas, like γδCTCL.

**Immunophenotyping Methodology**

45 skin lesions were derived from formalin fixed paraffin-embedded tissue and were stained at the Pathology Core Facility of Memorial Hospital. The antibodies for GATA3, Tbet, Bcl6 and FoxP3 (all SantaCruz) for immunohistochemistry were all validated with positive and negative controls. Expression was quantified for all four transcription factors as a percent-positive fraction among malignant T cells, which were defined morphologically by their nuclear atypia and features of epidermotropism or folliculotropism. In 7 SS samples, with verified circulating clonal CD4+CD7- cells, transcription factor expression was assessed in clonal T cells with multiparamateric intracellular flow cytometry. Peripheral blood mononuclear cells (PBMC) from these patients were previously isolated from whole blood samples using density gradient centrifugation (Ficoll-paque Plus, GE Healthcare) and cryopreserved in freezing medium containing 90% fetal bovine serum (FBS) and 10% dimethyl sulfoxide. Once thawed in warm complete medium (RPMI-10% FBS, 2%L-Glutamine, 1%Penicillin-Streptomycin), PBMC were rested for 30 minutes at 37ºC, washed twice in PBS and stained 30 minutes with a fixable yellow live/dead indicator on ice (ThermoFisher Scientific). Subsequently, samples were washed and stained for surface markers, with anti-CD3-PerCP-Cy5.5, anti-CD4-PE-Cy7 and anti-CD7-Alexa700 (BD Pharmingen). Samples were fixed and permeabilized with the eBioscience FoxP3/Transcription Factor kit (ThermoFisher Scientific), and stained for transcription actors with anti-Bcl6-PE, anti-Tbet-Alexa647, anti-GATA3-Alexa488 and anti-FoxP3-PE (BD Pharmingen). Samples were acquired on an LSR-II flow cytometer and analyzed on FlowJo v.9.7.6. Transcription factor expression was measured on live/CD3+/CD4+/CD7- malignant T cells and the quantification for all four transcription factors was calculated as the percent-positive fraction of cells compared to an FMO (fluorescence minus one) control.

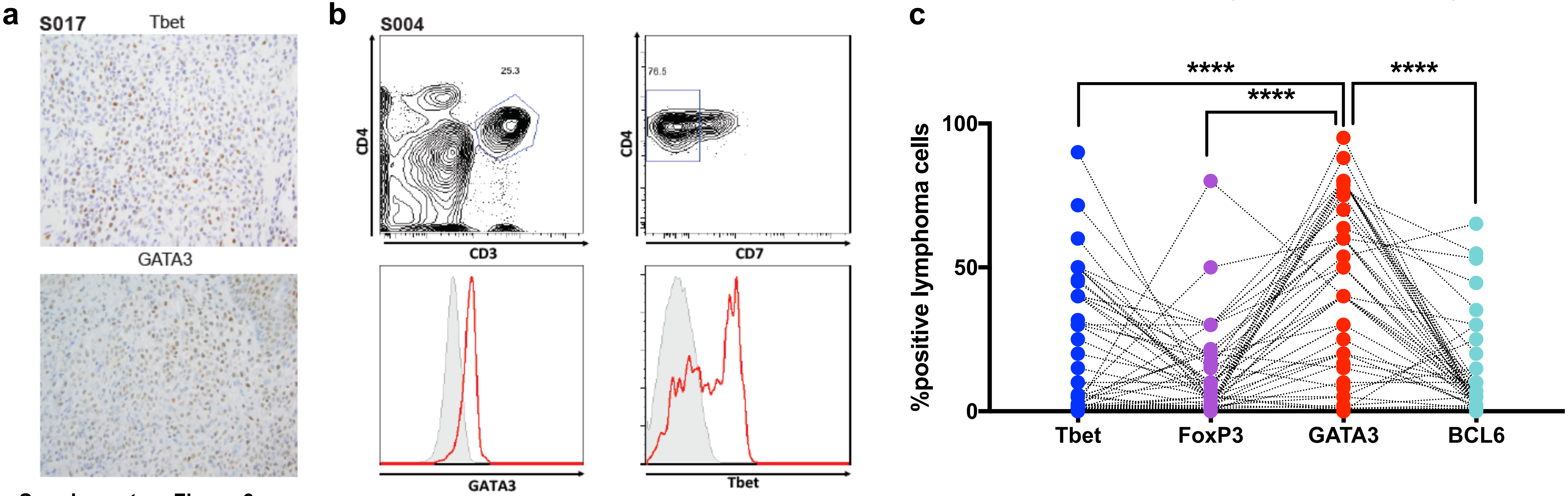


**Supplementary Figure 6**

Representative cases evaluated for GATA3 and Tbet expression by IHC and flow cytometry (**a** and **b**). Quantification of four Th transcription factors assessed by IHC and flow cytometry with heterogeneous patterns of expression. GATA3 was most highly expressed among all transcription factors (**c**, $p<0.001$)

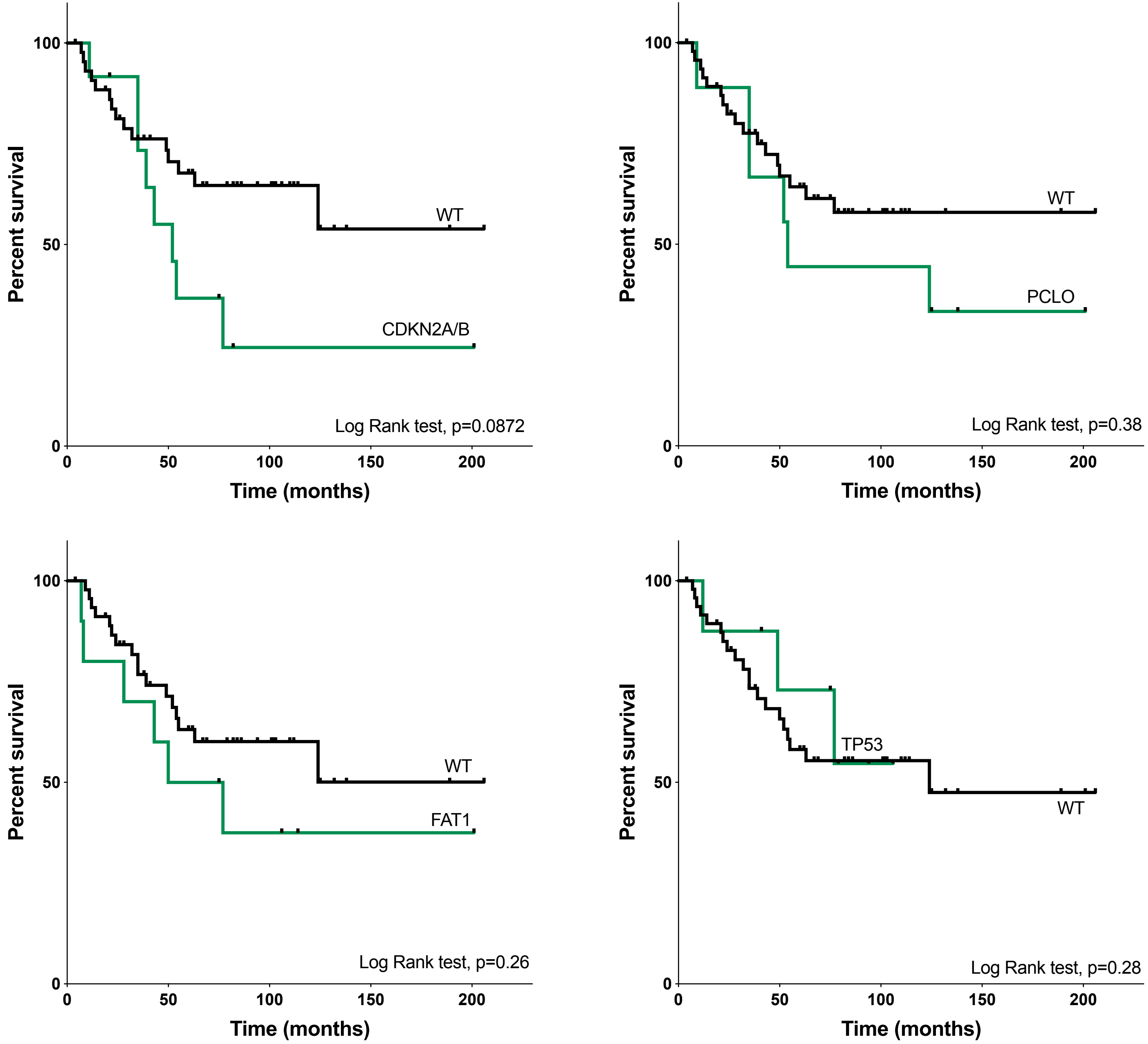


**Supplementary Figure 7**

Overall survival, univariate analysis per CDKN2A/B, PCLO, FAT1 and TP53 mutation status in non-eMF patients (Log Rank test).
