# Supplementary figures and images for: Targeted genomic analysis of cutaneous T cell lymphomas identifies a subset with aggressive clinicopathological features
Source: Blood Cancer J. 2020 Nov 9;10(11):116. doi: 10.1038/s41408-020-00380-5 (PMC7653963; doi:10.1038/s41408-020-00380-5)

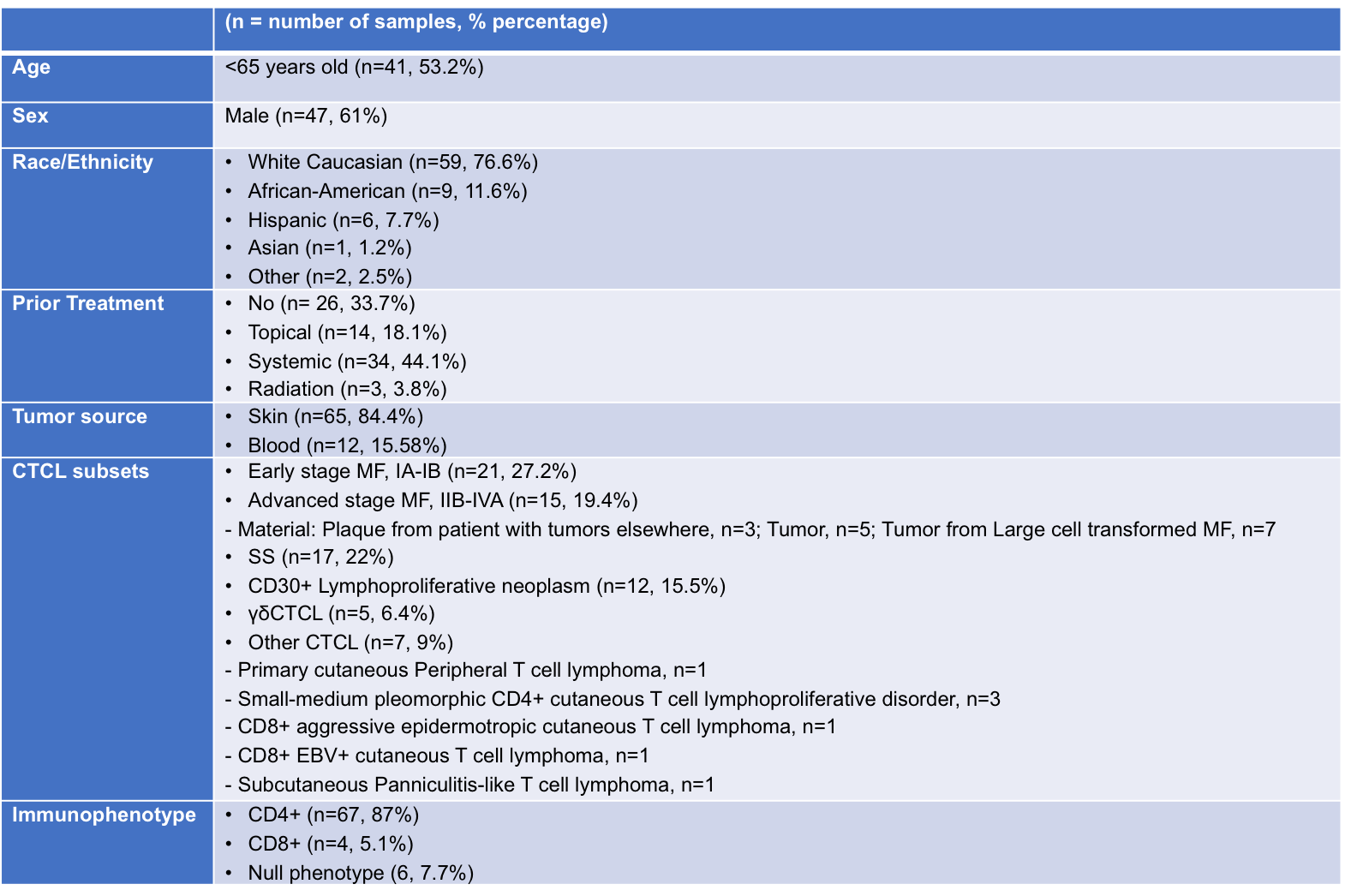


**Supplementary Table 1**

**Supplementary Table 1**

Supplement: Supplementary file 2 — Supplementary Table 1 [file 41408_2020_380_MOESM2_ESM.docx]
